# Supplementary material for: The Establishment and Optimization of a Chicken Primordial Germ Cell Induction Model Using Small-Molecule Compounds
Source: Animals (Basel). 2024 Jan 18;14(2):302. doi: 10.3390/ani14020302 (PMC10812757; doi:10.3390/ani14020302)
Supplement: Supplementary file 1 [file animals-14-00302-s001.zip › Supplementary material.pdf]

Supplementary material

Supplementary Figure legends

Supplementary Figure S1 Statistical analysis of G1/G2/S stage ratio during ESCs division.

Supplementary Figure S2 cESCs was induced into embryoid bodies by RA. A. The number of EBs during induction. (Data are shown as mean ± SEM, n = 3 independent experiments, ** $p < 0.01$, * $p < 0.05$, t test). B. qRT-PCR was used to detect the expression of marker genes of three germ layers *Pax6* (ectoderm), *Eomes* (mesoderm), *Viment* (endoderm) in ESCs, EBs, PGCs. (Data are shown as mean ± SEM, n =3 independent experiments, ** $p < 0.01$, * $p < 0.05$, one-way ANOVA).

Supplementary Figure S3 New factors can be used to optimize PGCs induction models *in vitro*. A. Correlation analysis between ESCs and PGCs samples. B, C. PPI interaction was used to analyze the interaction between different signaling pathways. D~H. Heat maps were used to analyze the expression of key genes during PGCs formation in different signaling pathways.

Supplementary Figure S4 New factors can be used to optimize PGCs induction models *in vitro*. Control settings and gating strategy for flow cytometry. a-e. Forward scatter and side scatter measurements are used to pre-gate the measured cell populations, then side scatter height and width measurements are used to gate individual cells, "negative" (A-B) and "positive" (C-E) stained cell populations. The border between is distinguished by the unstained sample in Figure 5D.

Supplementary Figure S5 Comparison of the migration efficiency between the isolated PGCs and the induced PGCLCs. A. Detection of PKH67 in the gonads of the recipient chicken embryos injected with the isolated PGCs and the induced PGCLCs. Scale bar: 60μm. B. Statistics on the migration efficiency of the isolated PGCs and the induced PGCLCs.
