# Supplementary material for: The Establishment and Optimization of a Chicken Primordial Germ Cell Induction Model Using Small-Molecule Compounds
Source: Animals (Basel). 2024 Jan 18;14(2):302. doi: 10.3390/ani14020302 (PMC10812757; doi:10.3390/ani14020302)
Supplement: Supplementary file 1 [file animals-14-00302-s001.zip › Supplementary Table S1.pdf]

Supplementary Table S1 GO terms related to stem cell differentiation

Id	Term	pVal	Gene
GO:2000736	regulation of stem cell differentiation	0	GDNF
GO:0048866	stem cell fate specification	0	SOX17
GO:2000741	positive regulation of mesenchymal stem cell differentiation	0	SOX5; SOX9
GO:1902459	positive regulation of stem cell population maintenance	0.000651441	ESRRB; LOC101748883; NANOG; PRDM14; Pou5f3
GO:2000737	negative regulation of stem cell differentiation	0.003812373	ESRRB; H1FOO; HES5(part)
GO:2000035	regulation of stem cell division	0.025542455	ESRRB; SOX17
GO:2000647	negative regulation of stem cell proliferation	0.025542455	KDF1; OVOL2
GO:1902455	negative regulation of stem cell population maintenance	0.025542455	LOXL2; WNT9B
GO:0048103	somatic stem cell division	0.025542455	TGFB2; ZFP36L2
GO:0048863	stem cell differentiation	0.045055186	A2M; GPM6A; HOXA7; LIF; LOC107053919 (part)
GO:0060218	hematopoietic stem cell differentiation	0.066187626	BATF; CHD2; MEOX1
GO:0097150	neuronal stem cell population maintenance	0.072138059	DLL1; HES5; LOC107057363; LOC419390; MMP24
GO:0048864	stem cell development	0.086745573	FGF2
GO:0072038	mesenchymal stem cell maintenance involved in nephron morphogenesis	0.086745573	WNT9B
GO:1902035	positive regulation of hematopoietic stem cell proliferation	0.155908611	KITLG; PDCD2
GO:0017145	stem cell division	0.18427156	ESRRB; GNL3; RGS10
GO:0005173	stem cell factor receptor binding	0.20915181	KITLG
GO:0072091	regulation of stem cell proliferation	0.20915181	SOX17
GO:0072089	stem cell proliferation	0.245629596	FGF2; TRIM71
GO:0035019	somatic stem cell population maintenance	0.315177703	CUL4A; SALL1; SALL4; SOX2; SOX9 (part)
GO:0071425	hematopoietic stem cell proliferation	0.340581553	CD34; MECOM
GO:0019827	stem cell population maintenance	0.664602719	EOMES; ESRRB; GNL3; LIF (part)
