# Supplementary material for: The Establishment and Optimization of a Chicken Primordial Germ Cell Induction Model Using Small-Molecule Compounds
Source: Animals (Basel). 2024 Jan 18;14(2):302. doi: 10.3390/ani14020302 (PMC10812757; doi:10.3390/ani14020302)
Supplement: Supplementary file 1 [file animals-14-00302-s001.zip › Supplementary Table S10.pdf]

Supplementary Table S10 qRT-PCR primers sequence of related genes

Gene	F (5'-3')	R (5'-3')
β-actin	ACCGCAAATGCTTCTAAAC	GACTGCTGCTGACACCTTC
Nanog	GTATGCAACCAGCTCACC	TAGTAGTGTCCGCACCTAAC
Lin28	CCCCAGCCTTGCCTTTATT	CGTCATTCACGCACATCCTA
Sox2	CATATGTAAGACAAAGGGG	AAGGTCCAGAATTTCTAATAA
Oct4	AAACGCAGGACCAGCATCG	AGCAGCCGCTTGCCTTTCT
Pax6	CCGACGGGATGTACGACAAG	CATCTGGGCCTCATCGGAAT
Eomes	ACAACGGTGAGAGAACCGTC	CTGCTGCACAGGAGTAACGA
Vimentin	GGACCTGCTGAATGTAAAGA	AGGTTGGAATAGGCATGTTA
Blimp1	AAGAATCTGGTGAAAGGGAG	GCAGTTTGATGCGTATT
Dazl	TACCCATTCGTCAACAACC	CACCTCCTTCACAGTACCATA
Cxcr4	ACTTGTCTTGCCTGGTCTA	TGATGCCAATGTAATACGG
Sox17	CGCTGCCCGCTATGATGCC	GCCGCTTGCGCTCGTCCTT
Cvh	AGCACAGGTGGTGAACGAA	TGCTGGTGGATGGTAGGTT
