# Supplementary material for: The Establishment and Optimization of a Chicken Primordial Germ Cell Induction Model Using Small-Molecule Compounds
Source: Animals (Basel). 2024 Jan 18;14(2):302. doi: 10.3390/ani14020302 (PMC10812757; doi:10.3390/ani14020302)
Supplement: Supplementary file 1 [file animals-14-00302-s001.zip › Supplementary Table S2.pdf]

Supplementary Table S2 GO terms related to germ cell formation

Id	Term	pVal	Gene
GO:0002467	germinal center formation	0	BCL6; KLHL6; MEF2C
GO:0001674	female germ cell nucleus	0	LHX8
GO:0030718	germ-line stem cell population maintenance	0	LOC107049165; PRDM14
GO:2000255	negative regulation of male germ cell proliferation	0.086745573	HPGDS
GO:0035234	ectopic germ cell programmed cell death	0.086745573	KITLG
GO:0019100	male germ-line sex determination	0.086745573	SOX9
GO:0051729	germline cell cycle switching, mitotic to meiotic cell cycle	0.20915181	LOC769510
GO:0002634	regulation of germinal center formation	0.20915181	MEF2C
GO:0007281	germ cell development	0.322301865	DND1; NANOS3; PRDM14; RGS10; TDRD1
GO:0001704	formation of primary germ layer	0.338693169	ATOH8
GO:0001673	male germ cell nucleus	0.568009514	TCFL5
GO:0097722	sperm motility	0	ASH1L
GO:0004766	spermidine synthase activity	0	SRM
GO:0097227	sperm annulus	0.007519789	SEPT12; SEPT2; SEPT6
GO:0097524	sperm plasma membrane	0.086745573	ABHD2
GO:2000360	negative regulation of binding of sperm to zona pellucida	0.086745573	ASTL
GO:1902093	positive regulation of flagellated sperm motility	0.086745573	PRDM14
GO:0035093	spermatogenesis, exchange of chromosomal proteins	0.155908611	PIWIL1; SYCP3
GO:0048240	sperm capacitation	0.198387624	ABHD2; CFTR; PCSK4; SLC26A6
GO:0008295	spermidine biosynthetic process	0.20915181	SRM
GO:0060468	prevention of polyspermy	0.338693169	ASTL
GO:0007342	fusion of sperm to egg plasma membrane involved in single fertilization	0.338693169	CD9
GO:0048515	spermatid differentiation	0.460549541	TMEM119

GO:0030317	flagellated sperm motility	0.495503889	CELF3; DDX4; DNAH5; INTS13; LRRC6 (part)
GO:0007286	spermatid development	0.551623589	CDYL; DHH; JAM3; KLHL26; KLHL32 (part)
GO:0007288	sperm axoneme assembly	0.562343392	NEURL1; PROCA1; SPAG6
GO:0007339	binding of sperm to zona pellucida	0.616679316	FETUB; PCSK4; ZP3; ZP3L1
GO:0097228	sperm principal piece	0.785508358	DNAH8; SPAG6
GO:0007283	spermatogenesis	0.998093464	ADAM28; ARID4B; ASZ1; AZIN2; BCL6 (part)
GO:0036126	sperm flagellum	0.999658567	ABHD2; AK1; C6H10orf107; DNAJB13; HEATR7B2L1 (part)
GO:0097225	sperm midpiece	0.999976247	HEATR7B2L1; HEATR7B2L2; (part)
