# Supplementary material for: The Establishment and Optimization of a Chicken Primordial Germ Cell Induction Model Using Small-Molecule Compounds
Source: Animals (Basel). 2024 Jan 18;14(2):302. doi: 10.3390/ani14020302 (PMC10812757; doi:10.3390/ani14020302)
Supplement: Supplementary file 1 [file animals-14-00302-s001.zip › Supplementary Table S3.pdf]

Supplementary Table S3 GO terms related to cell migration

Id	Term	pVal	Gene
GO:0061855	negative regulation of neuroblast migration	0	ABCC8
GO:1905322	positive regulation of mesenchymal stem cell migration	0	ACKR3
GO:0061870	positive regulation of hepatic stellate cell migration	0	ACTA2
GO:2000415	positive regulation of fibronectin-dependent thymocyte migration	0	ADAM8
GO:2000418	positive regulation of eosinophil migration	0	ADAM8
GO:0021801	cerebral cortex radial glia guided migration	0	ADGRG1
GO:0043615	astrocyte cell migration	0	APCDD1; CCAH221; CCLI7; CCLI8
GO:0061580	colon epithelial cell migration	0	ARSB
GO:0021853	cerebral cortex GABAergic interneuron migration	0	ARX; LHX6
GO:1905312	positive regulation of cardiac neural crest cell migration involved in outflow tract morphogenesis	0	BMP7
GO:2000473	positive regulation of hematopoietic stem cell migration	0	CCR2
GO:0035691	macrophage migration inhibitory factor signaling pathway	0	CD74
GO:0035692	macrophage migration inhibitory factor receptor complex	0	CD74
GO:0099606	microtubule plus-end directed mitotic chromosome migration	0	CENPE
GO:1904141	positive regulation of microglial cell migration	0	CSF1
GO:0021813	cell-cell adhesion involved in neuronal-glial interactions involved in cerebral cortex radial glia guided migration	0	DAB1
GO:0097477	lateral motor column neuron migration	0	DAB1; LHX1
GO:0003273	cell migration involved in endocardial cushion formation	0	DCHS1
GO:0036336	dendritic cell migration	0	DOCK8; EPS8
GO:0021885	forebrain cell migration	0	EMX2
GO:0003345	proepicardium cell migration involved in pericardium morphogenesis	0	FLRT3
GO:0003147	neural crest cell migration involved in heart formation	0	FOLR1
GO:0003253	cardiac neural crest cell migration involved in outflow tract morphogenesis	0	FOLR1; HAND2
GO:0021855	hypothalamus cell migration	0	FOXB1

GO:0061381	cell migration in diencephalon	0	FOXB1
GO:0097241	hematopoietic stem cell migration to bone marrow	0	GAS6; GPLD1
GO:0071674	mononuclear cell migration	0	LGALS3
GO:0071677	positive regulation of mononuclear cell migration	0	LGALS3
GO:2000403	positive regulation of lymphocyte migration	0	MADCAM1
GO:1905517	macrophage migration	0	MCOLN2
GO:0003259	cardioblast anterior-lateral migration	0	MESP1
GO:0060975	cardioblast migration to the midline involved in heart field formation	0	MESP1
GO:0022035	rhombomere cell migration	0	NRP1
GO:1904139	regulation of microglial cell migration	0	P2RY12
GO:0035793	positive regulation of metanephric mesenchymal cell migration by platelet-derived growth factor receptor-beta signaling pathway	0	PDGFA; PDGFB
GO:2000591	positive regulation of metanephric mesenchymal cell migration	0	PDGFB
GO:0021934	hindbrain tangential cell migration	0	PHOX2B
GO:1901166	neural crest cell migration involved in autonomic nervous system development	0	PHOX2B; SEMA3F
GO:0036486	ventral trunk neural crest cell migration	0	SEMA3F
GO:0030335	positive regulation of cell migration	9.78E-06	ADRA2A; ATP8A1; CCAH221; CCLI7; CCLI8 (part)
GO:0001755	neural crest cell migration	3.11E-05	ANXA6; ERBB4; GDNF; ISL1; KITLG (part)
GO:0010596	negative regulation of endothelial cell migration	0.003495186	ACVRL1; ADAMTS9; DLL4; LOC100858919; NR2F2 (part)
GO:0014911	positive regulation of smooth muscle cell migration	0.003766026	BCL2; CCL5; PDGFB; POSTN; SEMA6D (part)
GO:0030336	negative regulation of cell migration	0.003866281	ABHD2; ABHD6; ACVRL1; ADARB1; ARAP3 (part)
GO:0010595	positive regulation of endothelial cell migration	0.00432758	ADGRA2; ATOH8; CCBE1;

			FGF2; GATA3 (part)
GO:0021800	cerebral cortex tangential migration	0.007519789	ARX; LHX6; RELN
GO:0002687	positive regulation of leukocyte migration	0.010023702	ITGA2B; KITLG; MADCAM1; TNFRSF18
GO:0043542	endothelial cell migration	0.016208055	ADGRA2; CDH13; FAP; LOXL2; PLEKHG5 (part)
GO:0001764	neuron migration	0.022019035	ADGRL3; ASCL1; AUTS2; CCK; CDK5R2 (part)
GO:0021795	cerebral cortex cell migration	0.023045816	CX3CR1; EFHC1; EGFR; FGF13; PEX13
GO:0072676	lymphocyte migration	0.025542455	GATA3; TBX21
GO:1902766	skeletal muscle satellite cell migration	0.025542455	RHOC; SDC4
GO:0043534	blood vessel endothelial cell migration	0.026558105	ADAM8; PTK2B; SCARB1; VHL
GO:0008347	glial cell migration	0.026558105	CSPG4; NKX2-2; P2RY12; TGFB2
GO:0002042	cell migration involved in sprouting angiogenesis	0.02874501	EFNB2; EGR3; GPLD1
GO:0006929	substrate-dependent cell migration	0.02874501	FMNL1; PDGFB; VEGFC
GO:0014912	negative regulation of smooth muscle cell migration	0.02874501	IGFBP3; SEMA6D; TRIB1
GO:0072678	T cell migration	0.02874501	LOC107049666; MYO1G; S1PR1
GO:0043537	negative regulation of blood vessel endothelial cell migration	0.053782414	ACVRL1; AGTR2; RGCCL; VASH1
GO:2000406	positive regulation of T cell migration	0.066187626	ADAM8; APP; DOCK8
GO:2001223	negative regulation of neuron migration	0.066187626	ADGRG1; ERBB4; KIAA2022
GO:0042074	cell migration involved in gastrulation	0.066187626	CER1; SOX17; WNT11B
GO:0021799	cerebral cortex radially oriented cell migration	0.066187626	DIXDC1; LHX6; POU3F2

GO:1904754	positive regulation of vascular associated smooth muscle cell migration	0.072138059	DOCK4; IQGAP3; PDGFB; SSH1; XBP1
GO:2001222	regulation of neuron migration	0.072138059	FLRT2; IGSF10; SCRT2; SOX14; UNC5D
GO:0002523	leukocyte migration involved in inflammatory response	0.07961045	ADAM8; JAM3
GO:0010594	regulation of endothelial cell migration	0.07961045	BMPER; LOC112530215
GO:0051549	positive regulation of keratinocyte migration	0.07961045	FGF7; HBEGF
GO:0010631	epithelial cell migration	0.07961045	NANOS1; PKN3
GO:1901164	negative regulation of trophoblast cell migration	0.086745573	ARHGDIB
GO:0021831	embryonic olfactory bulb interneuron precursor migration	0.086745573	ARX
GO:0090132	epithelium migration	0.086745573	BVES
GO:2000417	negative regulation of eosinophil migration	0.086745573	CD300LG
GO:0035718	macrophage migration inhibitory factor binding	0.086745573	CD74
GO:0006931	substrate-dependent cell migration, cell attachment to substrate	0.086745573	CUZD1
GO:0051548	negative regulation of keratinocyte migration	0.086745573	EPPKL
GO:0051546	keratinocyte migration	0.086745573	FERMT1
GO:0022029	telencephalon cell migration	0.086745573	FOXB1
GO:0035701	hematopoietic stem cell migration	0.086745573	GPLD1
GO:0002686	negative regulation of leukocyte migration	0.086745573	HOXA7
GO:0003366	cell-matrix adhesion involved in ameboidal cell migration	0.086745573	LOC107049666
GO:0035046	pronuclear migration	0.086745573	LRMP
GO:0008078	mesodermal cell migration	0.086745573	MESP1
GO:0022028	tangential migration from the subventricular zone to the olfactory bulb	0.086745573	SLIT1
GO:0097324	melanocyte migration	0.086745573	ZEB2
GO:0016477	cell migration	0.111115651	ADAMTS12; ADGRG1; ARC; ASAP3; BLK (part)
GO:0010634	positive regulation of epithelial cell migration	0.138713148	CTSH; DOCK1; GLIPR2; PPM1F; PRKCE (part)

GO:0010633	negative regulation of epithelial cell migration	0.141001458	MARVELD3; PBLD; PTPN23; PTPRR
GO:0050900	leukocyte migration	0.144300037	ADD2; CD244; CD34; CD48; CD74 (part)
GO:0030334	regulation of cell migration	0.144300037	CCR2; DOCK10; ERBB4; FRMD5; LAMA2 (part)
GO:0090131	mesenchyme migration	0.155908611	ACTA2; ACTG2
GO:0051451	myoblast migration	0.155908611	MEGF10; SIX4
GO:2000405	negative regulation of T cell migration	0.20915181	APOD
GO:0060973	cell migration involved in heart development	0.20915181	BVES
GO:1990266	neutrophil migration	0.20915181	MCOLN2
GO:0014910	regulation of smooth muscle cell migration	0.20915181	PDGFA
GO:0021942	radial glia guided migration of Purkinje cell	0.245629596	CTNNA2; DAB1
GO:0043536	positive regulation of blood vessel endothelial cell migration	0.262014859	PDGFB; TMSB4X; TMSB4Y; VEGFC
GO:0090051	negative regulation of cell migration involved in sprouting angiogenesis	0.33587616	DLL4; KLF4; MEOX2
GO:0010762	regulation of fibroblast migration	0.33587616	PRR5L; RFFL; SDC4
GO:0010632	regulation of epithelial cell migration	0.338693169	ARSB
GO:0001667	ameboidal-type cell migration	0.338693169	CAP1
GO:0010764	negative regulation of fibroblast migration	0.338693169	CYGB
GO:0006930	substrate-dependent cell migration, cell extension	0.338693169	P2RY12
GO:0010763	positive regulation of fibroblast migration	0.434367549	DDR2; PRKCE
GO:0090050	positive regulation of cell migration involved in sprouting angiogenesis	0.460549541	HDAC9
GO:0022027	interkinetic nuclear migration	0.460549541	KIF1A
GO:1901165	positive regulation of trophoblast cell migration	0.460549541	SYDE1
GO:0010761	fibroblast migration	0.460549541	ZFAND5
GO:0007097	nuclear migration	0.673170094	FHOD1; SLIT1
GO:2001224	positive regulation of neuron migration	0.687118776	IL1R1; IL1RL2; SEMA3A
