# Supplementary material for: The Establishment and Optimization of a Chicken Primordial Germ Cell Induction Model Using Small-Molecule Compounds
Source: Animals (Basel). 2024 Jan 18;14(2):302. doi: 10.3390/ani14020302 (PMC10812757; doi:10.3390/ani14020302)
Supplement: Supplementary file 1 [file animals-14-00302-s001.zip › Supplementary Table S4.pdf]

Supplementary Table S4 GO terms related to Wnt signaling pathway

Id	Term	pVal	Gene
GO:0021881	Wnt-activated signaling pathway involved in forebrain neuron fate commitment	0	AXIN1
GO:0060823	canonical Wnt signaling pathway involved in neural plate anterior/posterior pattern formation	0	AXIN1
GO:0061290	canonical Wnt signaling pathway involved in metanephric kidney development	0	GATA3; LGR4
GO:2000057	negative regulation of Wnt signaling pathway involved in digestive tract morphogenesis	0	LOC395991; LOC417741; SFRP5
GO:0090082	positive regulation of heart induction by negative regulation of canonical Wnt signaling pathway	0	MESP1
GO:1901231	positive regulation of non-canonical Wnt signaling pathway via JNK cascade	0	NKD1
GO:2000054	negative regulation of Wnt signaling pathway involved in dorsal/ventral axis specification	0	SOST
GO:0003308	negative regulation of Wnt signaling pathway involved in heart development	0	SOX17
GO:0045813	positive regulation of Wnt signaling pathway, calcium modulating pathway	0	VANGL2
GO:1905438	non-canonical Wnt signaling pathway involved in midbrain dopaminergic neuron differentiation	0	WNT9B
GO:0017147	Wnt-protein binding	0.000571801	APCDD1; EGF; FZD1; FZD10; FZD2 (part)
GO:0042813	Wnt-activated receptor activity	0.003601693	EGF; FZD1; FZD10; FZD2; FZD4 (part)
GO:0035567	non-canonical Wnt signaling pathway	0.006150084	FZD1; FZD10; FZD2; FZD4; FZD5 (part)
GO:0060070	canonical Wnt signaling pathway	0.006434904	DIXDC1; DVL1; EGF; FZD1; FZD10 (part)

GO:2000096	positive regulation of Wnt signaling pathway, planar cell polarity pathway	0.025542455	ANKRD6; NKD1
GO:1905426	positive regulation of Wnt-mediated midbrain dopaminergic neuron differentiation	0.025542455	LMX1A; LOC101750514
GO:0061357	positive regulation of Wnt protein secretion	0.025542455	PTPN23; WLS
GO:0030111	regulation of Wnt signaling pathway	0.043404862	DCDC2; FOXL1; HNF1B; MDFIC; VANGL2
GO:0030178	negative regulation of Wnt signaling pathway	0.056938029	APCDD1; BARX1; DKK2; LOC395991; LOC417741 (part)
GO:0044332	Wnt signaling pathway involved in dorsal/ventral axis specification	0.07961045	EGF; FZD5
GO:2000052	positive regulation of non-canonical Wnt signaling pathway	0.07961045	LOC112532128; RSPO3
GO:0044336	canonical Wnt signaling pathway involved in negative regulation of apoptotic process	0.07961045	MITF; TCF7
GO:0038031	non-canonical Wnt signaling pathway via JNK cascade	0.086745573	MYOC
GO:0060828	regulation of canonical Wnt signaling pathway	0.129047475	AMER2; ANKRD10; FOLR1; FZD5; KREMEN1 (part)
GO:0090090	negative regulation of canonical Wnt signaling pathway	0.132146928	AMER2; ANKRD6; BICC1; DKK2; DRAXIN (part)
GO:0030177	positive regulation of Wnt signaling pathway	0.134733205	ATP6V1C2; DVL1; SALL1; SULF1; SULF2 (part)
GO:0007223	Wnt signaling pathway, calcium modulating pathway	0.198387624	FZD4; FZD5; LEF1; NFATC1
GO:2000051	negative regulation of non-canonical Wnt signaling pathway	0.20915181	SFRP4

GO:1904929	coreceptor activity involved in Wnt signaling pathway, planar cell polarity pathway	0.338693169	PTK7
GO:0016055	Wnt signaling pathway	0.456010391	AMER2; AMOTL2; APCDD1; CELSR2; CPE (part)
GO:1990909	Wnt signalosome	0.460549541	DVL1
GO:0060071	Wnt signaling pathway, planar cell polarity pathway	0.495503889	CELSR2; DVL1; FZD5; PRICKLE4; VANGL2 (part)
GO:0090263	positive regulation of canonical Wnt signaling pathway	0.753019153	DIXDC1; DKK2; DVL1; FAM53B; FGF2 (part)
