# Supplementary material for: The Establishment and Optimization of a Chicken Primordial Germ Cell Induction Model Using Small-Molecule Compounds
Source: Animals (Basel). 2024 Jan 18;14(2):302. doi: 10.3390/ani14020302 (PMC10812757; doi:10.3390/ani14020302)
Supplement: Supplementary file 1 [file animals-14-00302-s001.zip › Supplementary Table S5.pdf]

Supplementary Table S5 GO terms related to BMP signaling pathway

Id	Term	pVal	Gene
GO:1902813	negative regulation of BMP signaling pathway involved in determination of lateral mesoderm left/right asymmetry	0	CER1
GO:0071773	cellular response to BMP stimulus	0.002013323	ACVRL1; ADAMTS12; BMP6; BMP7; GATA3 (part)
GO:0030509	BMP signaling pathway	0.002318165	ACVRL1; BMP4; BMP6; BMP7; FAM83G (part)
GO:0030510	regulation of BMP signaling pathway	0.003409941	LOC395991; LOC417741; SFRP4; SFRP5; TFAP2B
GO:0070700	BMP receptor binding	0.007519789	BMP4; BMP6; BMP7
GO:0030514	negative regulation of BMP signaling pathway	0.007829194	BMPER; FSTL3; HTRA1; HTRA3; LRP2 (part)
GO:0030513	positive regulation of BMP signaling pathway	0.018443913	ACVRL1; CYR61; FBXL15; GATA6; HES5 (part)
GO:0035582	sequestering of BMP in extracellular matrix	0.086745573	CER1
GO:0061312	BMP signaling pathway involved in heart development	0.20915181	MSX2
GO:0098821	BMP receptor activity	0.460549541	ACVRL1
GO:0036122	BMP binding	0.658980698	CER1
