# Supplementary material for: The Establishment and Optimization of a Chicken Primordial Germ Cell Induction Model Using Small-Molecule Compounds
Source: Animals (Basel). 2024 Jan 18;14(2):302. doi: 10.3390/ani14020302 (PMC10812757; doi:10.3390/ani14020302)
Supplement: Supplementary file 1 [file animals-14-00302-s001.zip › Supplementary Table S6.pdf]

Supplementary Table S6 GO terms related to NOTCH signaling pathway

Id	Term	pVal	Gene
GO:0060853	Notch signaling pathway involved in arterial endothelial cell fate commitment	0	DLL1
GO:0035481	positive regulation of Notch signaling pathway involved in heart induction	0	MESP1
GO:1902367	negative regulation of Notch signaling pathway involved in somitogenesis	0	NRARP
GO:0007219	Notch signaling pathway	4.13E-05	AGXT; APP; ASCL1; CDH6; CDKN1B (part)
GO:0045746	negative regulation of Notch signaling pathway	0.007413254	ARRDC1; BMP7; C22H8ORF4; CBFA2T2; CHAC1 (part)
GO:0008593	regulation of Notch signaling pathway	0.010023702	ASCL1; DTX1; LFNG; POSTN
GO:0005112	Notch binding	0.022713872	C22H8ORF4; CHAC1; DLL1; DLL4; DNER (part)
GO:0035333	Notch receptor processing, ligand-dependent	0.025542455	DLL1; DLL4
GO:0007220	Notch receptor processing	0.053782414	DLL1; DLL4; DNER; PSEN2
GO:0061314	Notch signaling involved in heart development	0.20915181	DLL4
GO:0045747	positive regulation of Notch signaling pathway	0.21077859	ASCL1; DLL1; DLL4; HES5; JAG2 (part)
GO:0007221	positive regulation of transcription of Notch receptor target	0.733851748	PLXND1
