# Supplementary material for: The Establishment and Optimization of a Chicken Primordial Germ Cell Induction Model Using Small-Molecule Compounds
Source: Animals (Basel). 2024 Jan 18;14(2):302. doi: 10.3390/ani14020302 (PMC10812757; doi:10.3390/ani14020302)
Supplement: Supplementary file 1 [file animals-14-00302-s001.zip › Supplementary Table S7.pdf]

Supplementary Table S7 KEGG signaling pathway enrichment analysis

Term	pVal	Gene
MAPK signaling pathway	5.20E-05	CACNG4; TGFBR1; PDGFB; CACNA1B; DUSP1 (part)
TGF-beta signaling pathway	0.040321389	NOG; THBS1; TGFBR1; ID4; CDKN2B (part)
Wnt signaling pathway	0.006078829	FZD10; FZD4; FZD8; FZD2; FZD1 (part)
Notch signaling pathway	0.102007686	PSEN2; KAT2A; NOTCH1; LFNG; DLL1 (part)
Retinol metabolism	0.002039991	ALDH1A1; BCO1; ALDH1A2; ADH6; CYP1A2 (part)
PPAR signaling pathway	0.069521894	PPARD; SCD; RXRG; FABP7; PCK1 (part)
Hedgehog signaling pathway	0.273357314	SHH; IHH; PTCH1; GLI2; BCL2 (part)
ECM-receptor interaction	1.36E-09	LAMB2; THBS1; ITGB3; COL2A1; ITGB5 (part)
Cell adhesion molecules (CAMs)	2.45E-06	CLDN5; CLDN3; CDH5; SDC2; PTPRC (part)
D-Arginine and D-ornithine metabolism	0	DAO
Drug metabolism - cytochrome P450	1.31E-06	FMO3; ALDH1A3; GSTA4L; HPGDS; ADH6 (part)
Cell adhesion molecules (CAMs)	2.45E-06	CLDN5; CLDN3; CDH5; SDC2; PTPRC (part)
Metabolism of xenobiotics by cytochrome P450	2.79E-06	ALDH1A3; GSTA4L; HPGDS; ADH6; CYP1A2 (part)
Glycine, serine and threonine metabolism	5.22E-06	GLDC; GATM; CHDH; DMGDH; BHMT (part)
Focal adhesion	7.01E-06	ACTN1; VAV3; LAMB2; THBS1; PDGFB (part)
Cytokine-cytokine receptor interaction	5.49E-05	MSTN; TGFBR1; IL1RL1; TNFSF13B; IL16 (part)
Tryptophan metabolism	9.18E-05	CYP1A2; CYP1A1; AANAT; IL4I1; CCBL1 (part)
Calcium signaling pathway	0.000103374	EDNRA; EDNRB2; CACNA1B; CAMK2A; ATP2B1 (part)
Alanine, aspartate and glutamate metabolism	0.000132595	GAD2; GLUL; ASL1; ADSL; GPT2 (part)
Histidine metabolism	0.000343751	HAL; ALDH1A3; FTCD; UROC1; ASPA (part)
Tyrosine metabolism	0.000492853	ALDH1A3; DBH; TH; DCT; ADH6 (part)
Butanoate metabolism	0.00059007	GAD2; ABAT; HMGCLL1; BDH2; L2HGDH (part)

Nicotinate and nicotinamide metabolism	0.000602677	NT5M; NT5E; NT5C1B; CD38; BST1 (part)
Neuroactive ligand-receptor interaction	0.000699741	EDNRA; EDNRB2; CRHR1; CHRNA3; P2RX1 (part)
Caffeine metabolism	0.001967802	XDH; PNAT10; NAT; PNAT3
