# Supplementary material for: The Establishment and Optimization of a Chicken Primordial Germ Cell Induction Model Using Small-Molecule Compounds
Source: Animals (Basel). 2024 Jan 18;14(2):302. doi: 10.3390/ani14020302 (PMC10812757; doi:10.3390/ani14020302)
Supplement: Supplementary file 1 [file animals-14-00302-s001.zip › Supplementary Table S8.pdf]

Supplementary Table S8 GO terms related to MAPK signaling pathway

Id	Term	pVal	Gene
GO:0090080	positive regulation of MAPKKK cascade by fibroblast growth factor receptor signaling pathway	0	KLB
GO:0043408	regulation of MAPK cascade	0.002247	BMP4; BMP6; BMP7; C2orf88（part）
GO:1900745	positive regulation of p38MAPK cascade	0.008385	GADD45G; GDF6; GDF7; HAND2; MAPKKK3L; NCF1; SASH1
GO:1903753	negative regulation of p38MAPK cascade	0.086746	EZR
GO:0043410	positive regulation of MAPK cascade	0.103072	ADRB2; C1QTNF6; CCR2; CD74（part）
GO:0000165	MAPK cascade	0.124402	CCAH221; CCL5; CCLI7; CCLI8; CCR5（part）
GO:0032874	positive regulation of stress-activated MAPK cascade	0.154273	C26H1orf106; CARD9; MID1; STK25; TGFB2
GO:1900744	regulation of p38MAPK cascade	0.209152	HGF
GO:0000188	inactivation of MAPK activity	0.24563	DUSP4; DUSP8
GO:0000185	activation of MAPKKK activity	0.338693	GADD45G
GO:0000187	activation of MAPK activity	0.535128	C26H1orf106; CD74; CD81; CSPG4（part）
GO:0000186	activation of MAPKK activity	0.592304	EGFR; FGF2; MAP3K13; MAP3K15
GO:0043409	negative regulation of MAPK cascade	0.67317	ASH1L; DAG1
GO:0071848	positive regulation of ERK1 and ERK2 cascade via TNFSF11-mediated signaling	0	TNFRSF11A; TNFSF11
GO:0070374	positive regulation of ERK1 and ERK2 cascade	5.53E-05	ACKR3; ACTA2; APP; ARHGAP8; BMPER（part）
GO:0070373	negative regulation of ERK1 and ERK2 cascade	0.037334	CNKSR3; DUSP4; EZR; GPER1; KLF4（part）
GO:0070371	ERK1 and ERK2 cascade	0.053782	CTSH; SOX9; TNFSF11; ZFP36L2
GO:0070372	regulation of ERK1 and ERK2 cascade	0.085533	CYR61; EGFR; EPHA7; IL1B; ROS1; SYK
