# Supplementary material for: The Establishment and Optimization of a Chicken Primordial Germ Cell Induction Model Using Small-Molecule Compounds
Source: Animals (Basel). 2024 Jan 18;14(2):302. doi: 10.3390/ani14020302 (PMC10812757; doi:10.3390/ani14020302)
Supplement: Supplementary file 1 [file animals-14-00302-s001.zip › Supplementary Table S9.pdf]

Supplementary Table S9 GO terms related to cAMP signaling pathway

Id	Term	pVal	Gene
GO:0071320	cellular response to cAMP	0.001817262	AANAT; AKAP6; AQP1; AQP9 （part）
GO:0006198	cAMP catabolic process	0.023045816	PDE10A; PDE1A; PDE3B; PDE8A; PDE8B
GO:0030818	negative regulation of cAMP biosynthetic process	0.025542455	ADRA2A; GPR176
GO:2000480	negative regulation of cAMP-dependent protein kinase activity	0.026558105	PKIA; PKIB; PKIG; PRKAR1B
GO:0004862	cAMP-dependent protein kinase inhibitor activity	0.053782414	PKIA; PKIB; PKIG; PRKAR1B
GO:0019933	cAMP-mediated signaling	0.085532556	ADCY2; AKAP6; DGKQ; RAPGEF3; RIMS2; SOX9
GO:0030815	negative regulation of cAMP metabolic process	0.086745573	HTR1E
GO:0030822	positive regulation of cAMP catabolic process	0.086745573	RAPGEF3
GO:0051591	response to cAMP	0.129047475	DGKQ; HNF4A; JUN; JUND; PCK1; PTK2B; PTPRN
GO:0030814	regulation of cAMP metabolic process	0.155908611	DRD3; NPPC
GO:0030816	positive regulation of cAMP metabolic process	0.155908611	RAMP2; RAMP3
GO:0043951	negative regulation of cAMP-mediated signaling	0.18427156	APLNR; LPAR1; PDE3B
GO:2000479	regulation of cAMP-dependent protein kinase activity	0.20915181	LOC100858796
GO:0005222	intracellular cAMP activated cation channel activity	0.245629596	HCN3; HCN4
GO:0043949	regulation of cAMP-mediated signaling	0.245629596	PDE10A; RASD2
GO:0043950	positive regulation of cAMP-mediated signaling	0.257926279	ADRB1; PCK1; UCN
GO:0030552	cAMP binding	0.308163642	BVES; HCN3; HCN4; PDE10A; POPDC2; PRKAR1B; RAPGEF3
GO:0030819	positive regulation of cAMP biosynthetic process	0.414441037	ADRB1; GPER1; VIP
GO:0035497	cAMP response element binding	0.460549541	CREB3L3
GO:0005952	cAMP-dependent protein kinase complex	0.460549541	PRKAR1B

GO:0008603	cAMP-dependent protein kinase regulator activity	0.460549541	PRKAR1B
GO:0006171	cAMP biosynthetic process	0.997477652	ADCY2; ADCY5
