# Supplementary figures and images for: The Establishment and Optimization of a Chicken Primordial Germ Cell Induction Model Using Small-Molecule Compounds
Source: Animals (Basel). 2024 Jan 18;14(2):302. doi: 10.3390/ani14020302 (PMC10812757; doi:10.3390/ani14020302)

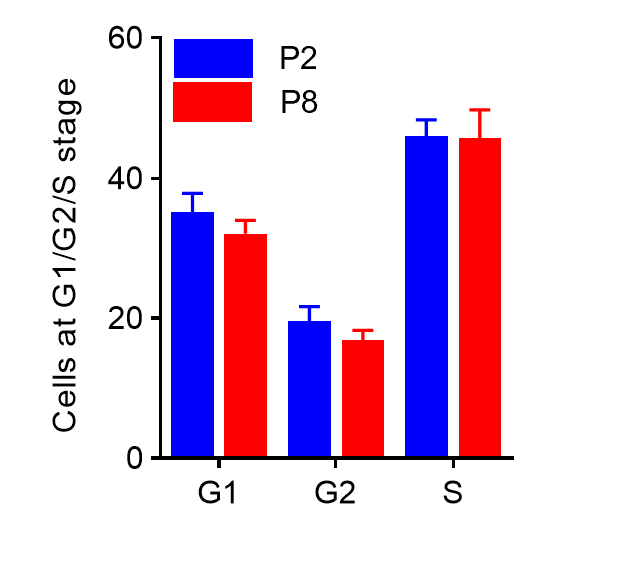

Supplement: Supplementary file 1 [file animals-14-00302-s001.zip › Supplementary Figure S1.tif]

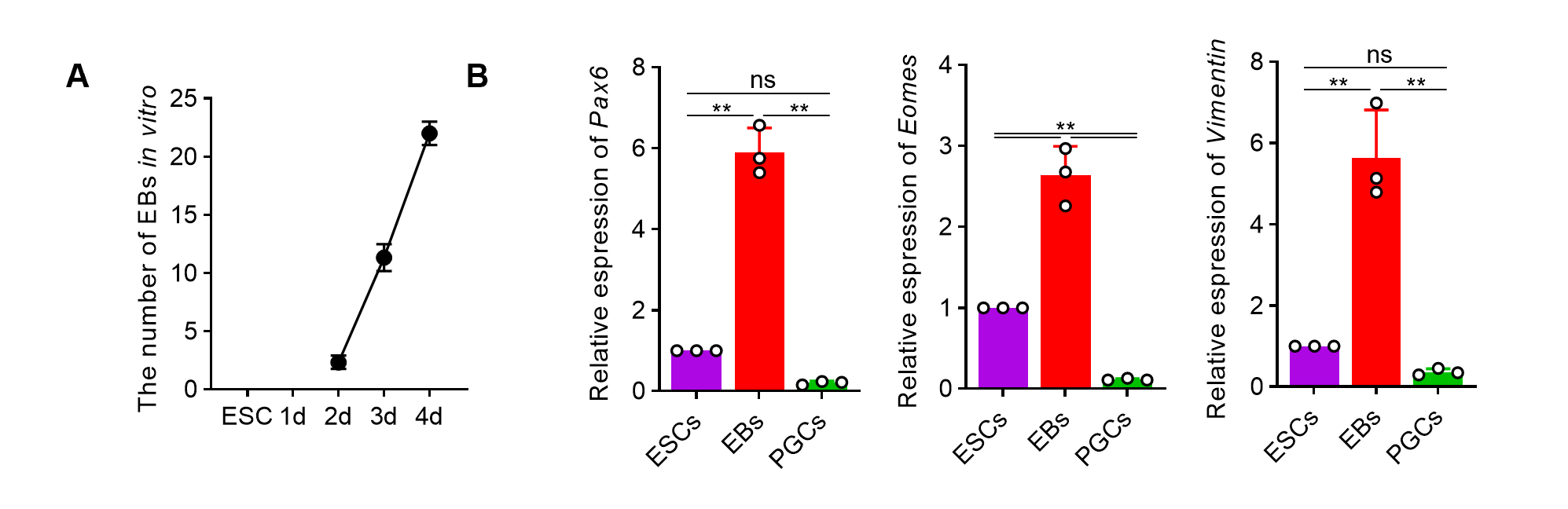

Supplement: Supplementary file 1 [file animals-14-00302-s001.zip › Supplementary Figure S2.tif]

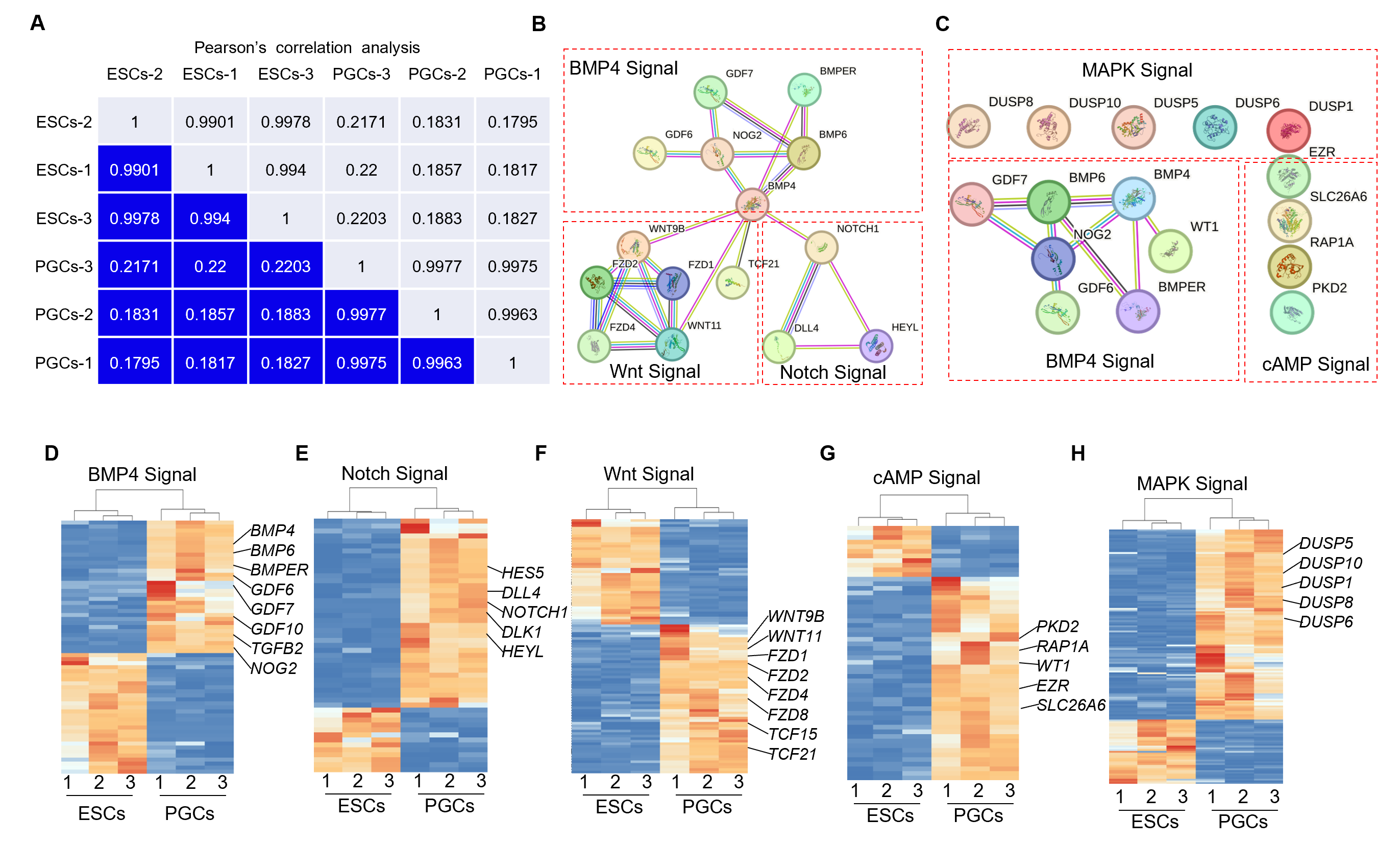

Supplement: Supplementary file 1 [file animals-14-00302-s001.zip › Supplementary Figure S3.tif]

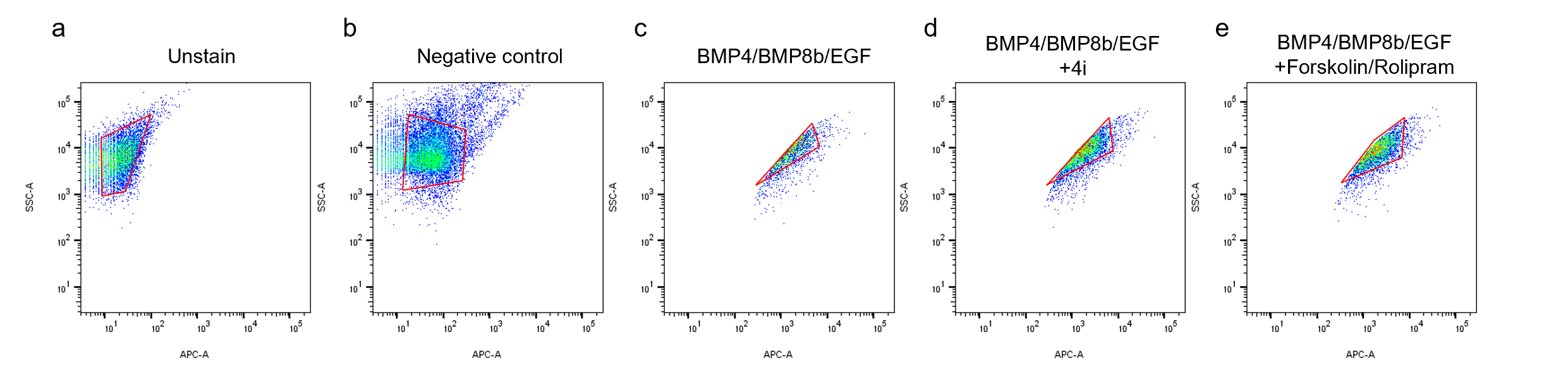

Supplement: Supplementary file 1 [file animals-14-00302-s001.zip › Supplementary Figure S4.tif]

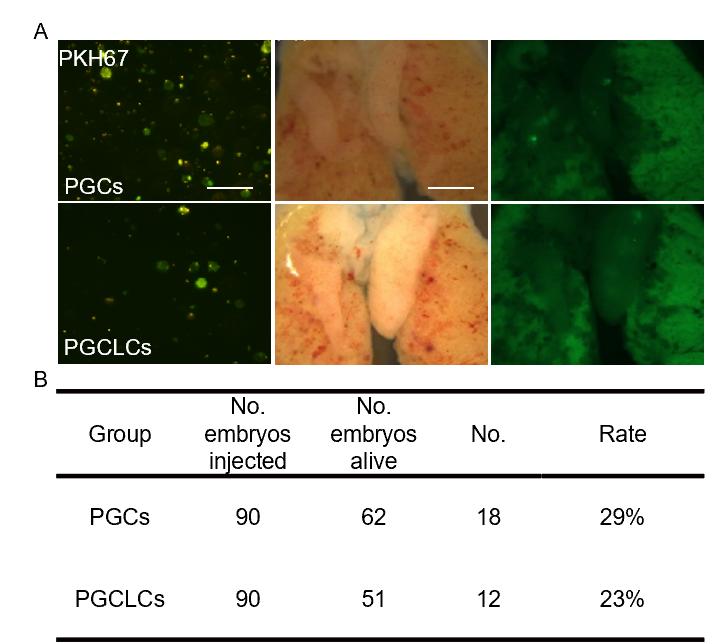

Supplement: Supplementary file 1 [file animals-14-00302-s001.zip › Supplementary Figure S5.tif]
